# Model‐informed unsupervised deep learning approaches to frequency and phase correction of MRS signals

**DOI:** 10.1002/mrm.29498

**Published:** 2022-11-11

**Authors:** Amirmohammad Shamaei, Jana Starcukova, Iveta Pavlova, Zenon Starcuk

**Affiliations:** ^1^ Institute of Scientific Instruments of the Czech Academy of Sciences Brno Czech Republic; ^2^ Department of Biomedical Engineering Brno University of Technology Brno Czech Republic

**Keywords:** deep learning, edited MRS, frequency correction, MR spectroscopy, phase correction

## Abstract

**Purpose:**

A supervised deep learning (DL) approach for frequency and phase correction (FPC) of MRS data recently showed encouraging results, but obtaining transients with labels for supervised learning is challenging. This work investigates the feasibility and efficiency of unsupervised deep learning–based FPC.

**Methods:**

Two novel deep learning–based FPC methods (deep learning–based Cr referencing and deep learning–based spectral registration), which use a priori physics domain knowledge, are presented. The proposed networks were trained, validated, and evaluated using simulated, phantom, and publicly accessible in vivo MEGA‐edited MRS data. The performance of our proposed FPC methods was compared with other generally used FPC methods, in terms of precision and time efficiency. A new measure was proposed in this study to evaluate the FPC method performance. The ability of each of our methods to carry out FPC at varying SNR levels was evaluated. A Monte Carlo study was carried out to investigate the performance of our proposed methods.

**Results:**

The validation using low‐SNR manipulated simulated data demonstrated that the proposed methods could perform FPC comparably with other methods. The evaluation showed that the deep learning–based spectral registration over a limited frequency range method achieved the highest performance in phantom data. The applicability of the proposed method for FPC of GABA‐edited in vivo MRS data was demonstrated. Our proposed networks have the potential to reduce computation time significantly.

**Conclusions:**

The proposed physics‐informed deep neural networks trained in an unsupervised manner with complex data can offer efficient FPC of large MRS data in a shorter time.

## 1 INTRODUCTION

In MRS, typically more transients are acquired and averaged to increase the low SNR.^1^ However, individual transients might have different frequency and phase shifts because of hardware imperfections, physiologic processes, or other instabilities.^2^, ^3^ Averaging transients without frequency and phase correction (FPC) would result in line‐broadening and lineshape imperfection of the combined MRS signal. Thus, FPC should be performed for each transient before averaging. It is even more critical to use accurate FPC while using spectral‐edited MRS^1^ methods to prevent artifacts caused by subtraction. Thus, FPC is a consensus‐recommended and effective step^4^ in MRS signal processing.

Several FPC approaches have been developed.^3^, ^5^, ^6^, ^7^, ^8^, ^9^, ^10^ The FPC methods can be classified into absolute and relative methods.^7^ Absolute approaches correct each individual transient absolutely, whereas relative methods align the transients to a reference signal. A commonly used approach for FPC is to use the water peak and read the phase and frequency from it.^6^, ^8^, ^11^ Another approach is to fit a certain metabolite peak to a model^12^ and then estimate the frequency and phase shifts from the model. One approach that has been proposed and evolved recently is spectral registration (SR).^3^, ^5^, ^9^, ^10^ Spectral registration fits each signal to a reference signal in the time domain through the adjustment of frequency and phase terms. Even though SR works very well for small shifts, it struggles with larger shifts and signals with low SNR. Modified versions of SR successfully addressed some of the mentioned problems.^5^, ^9^, ^10^ Most of these approaches are time‐consuming for large data sets, such as high‐resolution MRSI data sets, which may have thousands of spectra.

The recent success of deep learning (DL), one of the latest machine learning (ML) approaches, in a wide range of tasks, including the MR field,^13^, ^14^ suggests that it could also handle FPC. Recently, DL‐based solutions have been proposed for metabolite quantification in the frequency domain,^15^, ^16^ detecting and removing ghosting artifacts,^17^ FID reconstruction,^18^ automatic peak picking,^19^ enhancement of MRSI spatial resolution,^20^ and identifying and filtering out poor‐quality spectra.^21^ It has been shown that DL can also be used for FPC^7^, ^22^ and could speed up FPC once it has been successfully trained. This approach, using two separate networks in sequence to estimate frequency and phase, showed encouraging results. The first network was trained for frequency‐shift estimation using the magnitude of frequency‐shifted and phase‐shifted spectrum as the input and the known frequency shift as the output. Subsequently, the second network was trained for phase shift estimation using real parts of the frequency‐corrected spectrum as the input and phase shift as the output. In this approach, any error in the first step (frequency correction) may bias the phase shift estimation. Training two networks is a computationally expensive task. Moreover, the networks were trained in a supervised manner using simulated data. Any discrepancy between the in vivo and the simulated spectra may result in errors in frequency and phase shift estimation. The true output values are unknown in MRS data, and obtaining hundreds of spectra with labeled frequency and phase shifts is almost infeasible. Unsupervised learning may eliminate the drawbacks of supervised learning.

Frequency and phase correction is traditionally described by adjusting two parameters (frequency and phase). Therefore, it is natural to expect that the variability of all the signals in the set acquired for SNR improvement should have a very low‐dimensional representation. One of the methods for nonlinear dimensionality reduction is manifold learning, which assumes that the available high‐dimensional data vectors are embedded in low‐dimensional manifolds.^23^, ^24^ These low‐dimensional manifolds can be learned by deep autoencoders (DAEs),^25^ which automate feature extraction by merging all relevant data into a cohesive framework. A DAE with a common architecture^13^, ^25^ is not able to learn to estimate the frequency and the phase shift of a transient, as the features in a low‐dimensional space might not be readily interpretable. Therefore, a DAE can be redesigned to have two functions: a function for nonlinear mapping between the input and certain features (frequency and phase shifts) in a two‐dimensional space, and another function, for reconstructing the input from those features. Accordingly, we designed a DAE network that can learn in an unsupervised manner to estimate the frequency and phase shifts of MRS data. The proposed method takes advantage of the parametric analytical approach and embeds it into the DAE to estimate the frequency and phase shifts of a transient.

The proposed network was trained and validated using a simulated data set in which ground‐truth knowledge was available and evaluated using phantom and in vivo MEGA‐edited MRS data obtained from the publicly accessible Big GABA repository.^26^, ^27^ The FPC performance of our proposed network was compared with the commonly used FPC methods, namely, SR,^3^ spectral registration over a limited frequency range (SRF),^3^ frequency domain correlation,^5^ frequency domain correlation over a limited frequency range,^5^ creatine referencing (CrR),^12^, ^28^ as well as supervised deep‐learning approaches^7^, ^22^ in terms of precision and time efficiency.

## 2 METHODS

### 2.1 Data normalization

In contrast to the application of DL in machine vision or speech recognition, where the input data can be normalized by a nonlinear transform, MRS signals must be normalized by a linear transform. In this study, each complex signal S(t) was rescaled as

(1) $$S{\left(t\right)}_{\text{normalized}}=\frac{S\left(t\right)}{\max_{\tau }\left(\left|S\left(\tau \right)\right|\right)}.$$

A similar approach could be dividing the signal by the absolute value of the first point of the signal, but it would be less generally applicable because the initial point can be influenced by the filtering processes in the receive chain,^29^ or the maximum may occur later in an echo or with coupled resonances.

### 2.2 Data augmentation

It is known that the sufficient size and diversity of data are important factors for the effectiveness of most DL models.^13^ However, having rich and sufficient data sets is rare^30^ in the field of MRS and MRSI. Data augmentation is a viable option, which simulates credible data by minor alterations to data in a small existing data set. Data augmentation used in computer vision applications to reduce the generalization error of models^13^ applies to flips, translations, and rotations,^13^ which would be meaningless in spectroscopy. To generate credibly varied FID signals, we chose a set of physics‐informed alterations, simulating the practical data variability:

1. Frequency shift
2. Phase shift
3. Apodization (line broadening)
4. Amplitude change
5. Adding noise
6. Adding a nuisance peak (residual water and lipids)

### 2.3 Data sets

#### 2.3.1 Simulated data set

A simulated data set was used in in silico ground‐truth information for evaluating the performance of our proposed networks and for comparison with the commonly used FPC methods. The simulated data set was obtained by alternating a single MR signal acquired from a rat brain as described subsequently.

A single‐voxel spectroscopy (SVS) MR in vivo signal was acquired from a rat's right hippocampus (256 transients, voxel size = 1.5 × 1.5 × 4 mm^3^) in a 9.4T small animal MR system (Bruker BioSpin MRI, Ettlingen, Germany) using a PRESS sequence (spectral width = 4400 Hz, 2048 points, TE = 16.5 ms, TR = 2500 ms) with water and outer‐volume suppression by VAPOR.^31^ The signal (further referred to as the basis signal $S_{\text{basis}}\left(t\right)$) was created after transients were corrected for $B_{0}$ instability due to eddy currents as well as $B_{0}$ drift, and averaged using Bruker proprietary software, Paravision.

All experiments were approved by the Czech Governmental Animal Care Committee, in compliance with Czech Animal Protection.

The simulated data set, containing 24 000 artificial signals, was generated from $S_{\text{basis}}\left(t\right)$ by an augmentation procedure. The basis signal was multiplied by factors drawn from a normal distribution with a mean of 1 and a SD of 0.1. Then a set of lipid nuisance peak and a set of unstable residual water nuisance peak, generated using Equation 2 (parameters are listed in Supporting Information Table S1), were added to signals, randomly and independently:

(2) $$S{\left(t\right)}_{\text{nuisance peak}}=A_{a}e^{-d_{a}t}e^{-i\left(2\pi f_{a}t+\phi_{a}\right)},$$

where $i=\sqrt{-1}$; t is a vector of time points; and $A_{a},d_{a},f_{a}$, and $\phi_{a}$ are the amplitude, the damping factor, the precession frequency, and the phases of the nuisance peak, respectively. Signals containing the lipid peak, the unstable residual water peak, and both peaks were labeled as LC, UW, and UW & LC, respectively.

All artificial signals were further apodized by normally distributed random dampings corresponding to Lorentzian linewidths with a mean of 2 Hz and a SD of 0.2 Hz. Then, uniformly distributed artificial frequency and phase offsets in the range of −20 to 20 Hz and − 90° to 90°, respectively, were applied to signals. Before the normalizing step, the SNR of signals (time origin magnitude to noise SD) was set in the range of approximately 9 to 27 by introducing random complex Gaussian white noise. Signals in the simulated data set were shuffled randomly, and 90% of the data set was allocated to the training subset: 9% for the validation subset, and the remaining 1% for the test subset.

#### 2.3.2 Phantom data set

Phantom data were used to assess the performance of our methods in the absence of a large number of training data measured during temperature‐dependent changes in the $B_{0}$ field.

An SVS MR signal was acquired in a phantom of known metabolite concentrations (N‐acetylaspartate 17.5 mmol/L, glutamate 26.2 mmol/L, myo‐Inositol 17.5 mmol/L, creatine [CR] 12.7 mmol/L, taurine 10.1 mmol/L; 2048 transients, voxel size = 3 × 3 × 3 mm^3^) in a 9.4 T small animal MR system (Bruker BioSpin MRI, Ettlingen, Germany), while the temperature of the phantom was altered between 35°C and 40°C and the frequency adjustment of the scanner was switched off, using PRESS sequence (spectral width = 4400 Hz, 2048 points, TE = 16.5 ms, TR = 2500 ms) with water and outer‐volume suppression by VAPOR.^31^

The phantom data set, containing 24 000 artificial signals, was generated as follows: We selected 400 (out of 2048) of the acquired transients as basis signals, randomly; then, a subset of 60 signals was generated from each basis signal by the same augmentation procedure as used for the simulated data set except that the basis signal was not multiplied by factors, the nuisance peaks were not added, and the SNR of signals was set in the range of about 7 to 70. Finally, all subsets were stacked together to create the final training data set. The rest of the 1648 transients were used as an unseen test subset.

#### 2.3.3 Big GABA data set

Data from the public repository Big GABA^26^, ^27^ were used to demonstrate the applicability of the proposed method on FPC of edited in vivo signals.

We selected 48 GABA‐edited MEGA‐PRESS subsets (subjects) acquired on Siemens scanners from four different sites (S1, S5, S6, and S8 [all that we found public and readable]; 3T field strength; spectral width = 4000 Hz, 4096 points, TE = 68 ms; ON/OFF editing pulses = 1.9/7.46 ppm; editing pulse duration = 15 ms, TR = 2000 ms; 320 averages; 30 × 30 × 30 mm^3^; and medial parietal lobe voxel) because Siemens data^3^ had greater median within‐participant SD of estimated phase offsets and larger variance of lipid contamination than data from Philips and GE, and relatively high average frequency offsets, which are undesirable conditions for conventional FPC methods.^5^

We allocated 40 of 48 selected in vivo subsets (15 360 transients) to the training subset (12 800 transients), and the rest of the subsets (2560 transients) were used as an unseen test subset.

### 2.4 Deep model

#### 2.4.1 The DAE proposed for deep learning–based peak referencing

The DAE is a type of deep artificial neural network that is created to learn the coding of data in an unsupervised manner. The fundamental underlying concept of autoencoders is to use the input data as the target (ie, attempting to reconstruct the input data in the output layer).^13^ Typically, a DAE consists of two parts: an encoder and a decoder.

**FIGURE 1:**
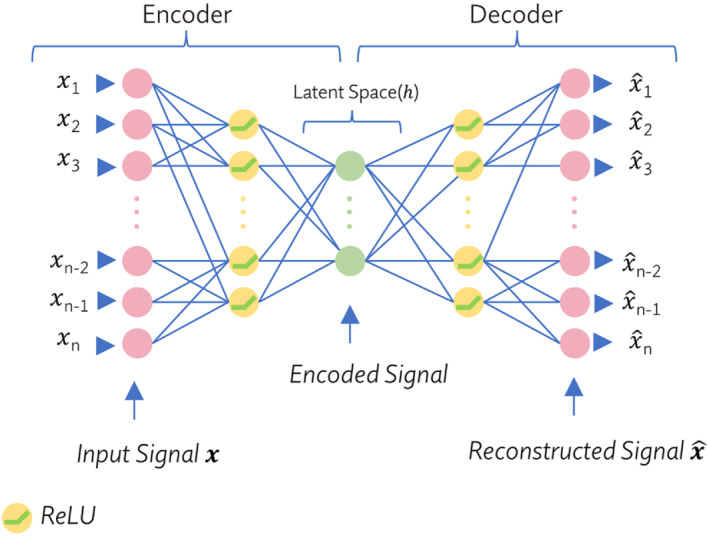
Illustration of a common deep autoencoder (DAE) architecture with multiple nonlinear hidden layers composed of rectified linear units (ReLUs) and fully connected (FC) layers to elicit nonlinear features of the data

Figure 1 illustrates the most common architecture of a DAE. The encoder function h=f(x) maps the n‐dimensional input vector ($x\in R^{n}$) to the $n^{\prime }$‐dimensional latent vector ($h\in R^{n^{\prime }}$), while the decoder function $\hat{x}=g\left(h\right)$ aims to reconstruct the n‐dimensional output vector ($\hat{x}\in R^{n}$) from the latent space representation. The mathematical expression of a DAE can be written as follows:

(3) $$\hat{x}=g\left(f\left(x;\theta_{e}\right);\theta_{d}\right),$$

where $\theta_{e}$ and $\theta_{d}$ are the parameters set of encoder and decoder layers, respectively.

**FIGURE 2:**
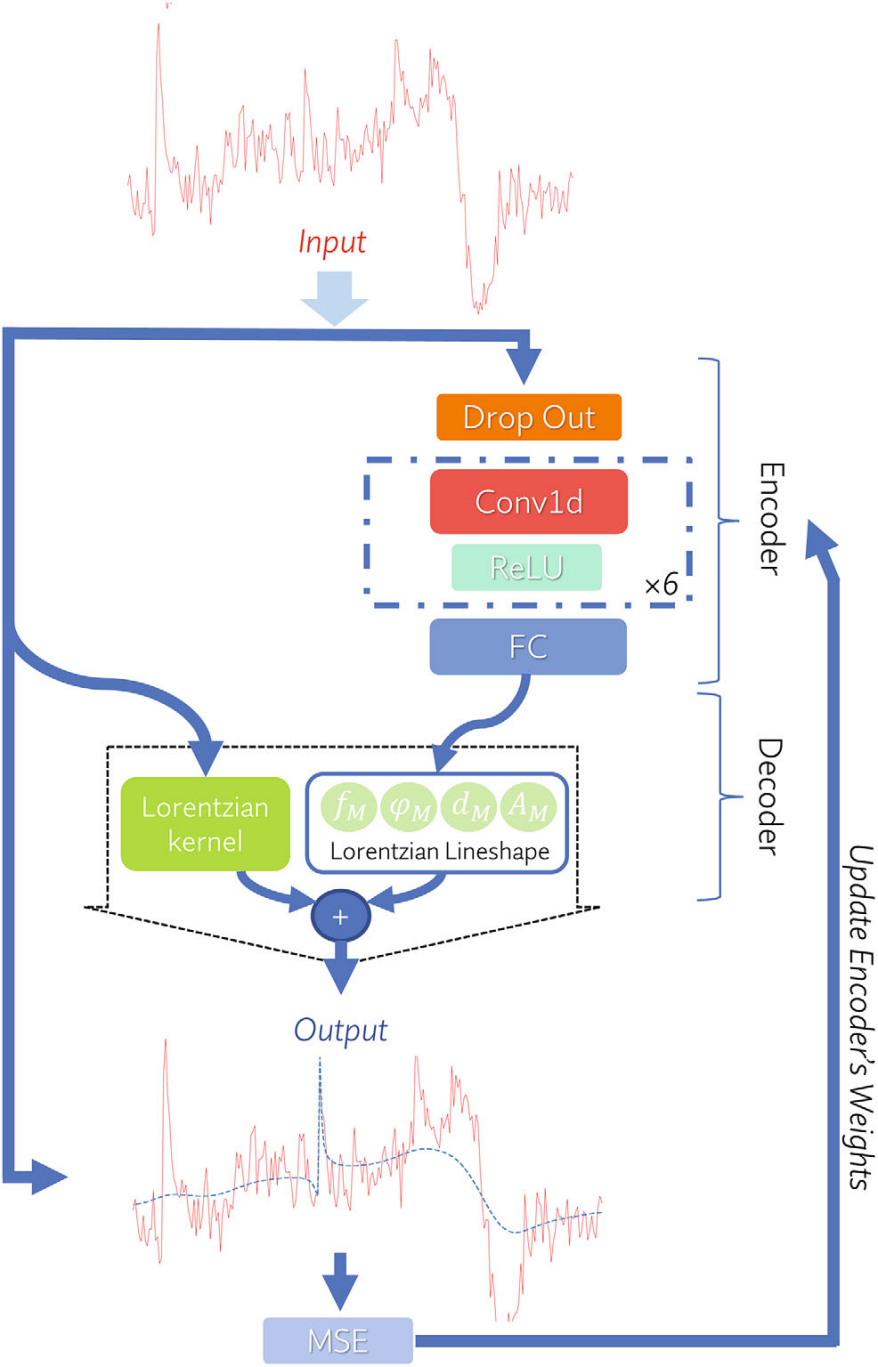
Illustration of the proposed convolutional encoder/model‐decoder for the deep learning–based Creatine referencing (dCrR) method. The network's input is a complex signal in the time domain, which is fed to the encoder. The encoder consists of a dropout layer, six convolutional blocks, and an FC layer (see details in Supporting Information Table S2). A convolutional block (dashed square) consists of a one‐dimensional convolution (Conv1d) layer followed by a ReLU layer. The model‐decoder of the dCrR (Equation 4) reconstructs the output signal. The DAE was trained to encode the input vector in the time domain into parameters that can be used to reconstruct the output vector in the time domain. The proposed network is trained by minimizing the mean square error (MSE) between the input and the output. The input and output signals are depicted in the frequency domain for the sake of easier understanding. Abbreviations: $A_{M},\text{amplitude};d_{M},\text{damping factor};f_{M}$, resonance frequency; $\phi_{M}$, zero‐order phase of the Lorentzian lineshape

For FPC, the latent space representation must be interpretable parameters, such as frequency and phase shifts. Frequency and phase shifts can be estimated by fitting a certain metabolite peak to a model, such as a Lorentzian lineshape. Then, the frequency and phase shifts can be read from the model. To this end, we proposed a convolutional encoder/model‐decoder^15^ architecture. Our proposed DAE has a conventional encoder consisting of a pipeline of a dropout layer,^32^ convolutional layers,^33^, ^34^ fully connected layers,^13^, ^34^ and rectified linear unit layers,^34^ which encode a complex input signal into a latent space, and a decoder that reconstructs a Lorentzian lineshape of a certain peak in the input signal using the latent space parameters. The proposed DAE architecture is depicted in Figure 2. Because it has been shown that using complex‐valued data improves the robustness and efficiency of fitting MRS data,^35^ the input and output of the proposed DAE were set to be complex signals in the time domain.

**TABLE 1:** Comparison of our proposed method with existing commonly used FPC methods for the simulated data set

		dSR	dSRF	SR	SRF	dCr	dCrF	Cr	Corr	CorrF	Tapper et al	Ma et al
Precision of frequency estimation (Hz)	All samples	1.02	0.73	1.57	11.46	0.94	0.95	7.92	10.70	11.33	**0.71**	0.99
	Samples without nuisance peaks	1.00	0.69	1.01	11.02	0.93	0.90	9.62	9.98	9.89	**0.56**	0.85
	Samples with nuisance peaks	0.97	**0.73**	1.57	11.68	0.94	0.97	6.51	11.07	11.96	0.78	1.06
Precision of phase estimation (°)	All samples	5.87	4.89	7.44	15.92	6.62	6.79	32.34	16.87	17.46	6.78	**4.29**
	Samples without nuisance peaks	4.16	3.34	**2.61**	16.75	6.89	7.00	38.80	16.62	16.46	4.64	4.24
	Samples with nuisance peaks	5.75	5.52	8.50	15.34	6.35	6.52	26.68	17.02	18.07	5.89	**4.32**
SI	All samples	0.44	**0.44**	0.44	0.32	0.42	0.42	0.36	0.34	0.32	0.44	0.44
Cr_linewidth_ (Hz)	Averaged signal	10.38	10.22	10.77	15.34	**10.14**	10.17	10.71	19.14	20.55	10.45	10.53
Training time (min) CPU	All samples	**127**	138	—	—	328	354	—	—	—	—	—
Training time (min) GPU	All samples	**2.26**	7.8	—	—	8.4	7.3	—	—	—	20.8	29.64
Training time (min)	All samples	**4.53**	10.6	—	—	11.1	11.2	—	—	—	—	—
Google Colaboratory												
Processing time (ms) CPU	Per signal	**3**	**3**	57	71	**3**	**3**	53	33	29	15	15
Processing time (ms) GPU	Per signal	**0.1**	**0.1**	—	—	0.2	0.2	—	—	—	0.6	0.6

*Note*: Bold text indicates the best performance in each metric. The SI of the test subset without FPC was 0.14. Precision: SD of the difference between the estimated and the true shift.

Abbreviations: Corr, frequency domain correlation; CorrF, frequency domain correlation over a limited frequency range; CPU, central processing unit; Cr_linewidth_, linewidth of Cr peak at 3 ppm; CrR, creatine referencing; GPU, graphics processing unit; SI, similarity index; SRF, SR over a limited frequency range.

Time‐domain fitting of a single Lorentzian lineshape to a signal with several peaks using our proposed network is a challenging task in which the optimization algorithm aims to increase the linewidth to decrease the error. Previous studies^3^, ^12^, ^28^ addressed these problems by fitting a signal in the frequency domain over a limited range and including a linear baseline in their model. We found that adding a rough estimate of a baseline, obtained by apodizing the input signal with Lorentzian kernel with a large linewidth, into the reconstruction function improves our fitting. Hence, the decoder part combines a mathematical model (Lorentzian lineshape) and the input signal, *x*, apodized with the Lorentzian kernel. The mathematical expression of the decoder can be written as follows:

(4) $$g\left(x;A_{M},d_{M},f_{M},\phi_{M},t\right)=A_{M}e^{-d_{M}t}e^{-i\left(2\pi f_{M}t+\phi_{M}\right)}+x\left(t\right)e^{-d_{L}t},$$

where $A_{M},d_{M},f_{M}$, and $\phi_{M}$ are the amplitude, the damping factor, the resonance frequency, and the zero‐order phase of the selected Lorentzian lineshape, respectively, and $d_{L}$ is the linewidth of the Lorentzian kernel. Experimentally, $d_{L}$ was set to 500 Hz in this study.

Training our proposed network is an unsupervised learning task that does not require ground‐truth frequency and phase shifts and can be done by minimizing the differences between the original input and the consequent reconstruction. In each iteration step of training, the parameters of the encoders are adjusted according to the gradient of the loss function with respect to the given parameters of the Lorentzian lineshape ($A_{M},d_{M},f_{M},\text{and}\phi_{M}$).

In this study, Cr peak at 3.027 ppm was selected to be fitted by a Lorentzian line shape in the decoder. The FIDs were truncated to the initial 512 points for limiting the contribution of noise, which typically predominates in the later part of FIDs. Then, the truncated FIDs were used as inputs to the network. After training, the encoder of the proposed DAE (deep learning–based creatine referencing [dCrR]) was detached from the network and used to estimate the frequency and phase of the Cr peak in a test transient. Then the estimates were used for frequency and phase correction of the test transient. The pipeline of FPC of GABA‐edited MEGA‐PRESS transients is provided in Supporting Information Figure S4.

#### 2.4.2 The DAE proposed for deep learning–based SR

With a simple modification, the proposed approach could estimate the relative frequency and phase shifts by fitting each signal to a reference signal and be applicable to various MRS experiments. In other words, the SR method could be used in our proposed encoder/model‐decoder network. This modification is referred to as deep learning–based spectral registration (dSR). For dSR, the mathematical expression of the decoder can be written as follows:

(5) $$g\left(f_{M},\phi_{M},t\right)=R\left(t\right)e^{-i\left(2\pi f_{M}t+\phi_{M}\right)},$$

where $f_{M}$ and $\phi_{M}$ are the frequency and the phase shifts of the input signal with respect to the reference scan R(t). Thus, in the dSR, the encoder estimates two parameters (relative phase and frequency shifts) instead of estimating the four parameters of a lineshape. The proposed DAE architecture for dSR is depicted in Supporting Information Figure S5. We proposed a new ML‐based algorithm for finding the reference signal in which the k‐means algorithm^36^ was used to cluster magnitude‐valued signals in the training set. The number of clusters was set to two: one for samples with nuisance peaks and another for samples without nuisance peaks. k‐Means algorithm is an unsupervised method; thus, the clusters are not identified. Based on prior testing, samples containing nuisance peaks have a higher intensity than samples without nuisance peaks in the initial points of their mean of the samples in the cluster. Therefore, the first 10 points of each mean of samples in the cluster were averaged, and the cluster with a lower average was identified as the cluster containing samples without nuisance peaks; then, the signal with the highest SNR from this cluster was selected as the reference scan (see details in Supporting Information Text S2 and Figure S6).

#### 2.4.3 Training the proposed DAEs over a limited frequency range

The MRS signals may have unstable frequency components. Because all frequency components are present at all time points, unstable frequency components may bring errors into our proposed method. To avert this situation, the proposed architectures (dCrR and dSR) were trained over a limited frequency range (2.5 to 3.5 ppm) (referred to as dCrRF [deep learning‐based creatine referencing over a limited frequency range] and dSRF [deep learning–based spectral registration over a limited frequency range]). Note that the input of the encoder and the output of the model‐decoder were still in the time domain, and the discrepancy between fast Fourier transformation of the input and output was calculated over a limited frequency range.

### 2.5 Implementation details and training

All steps were run on a computer with a dual EPYC 7742 (2 × 64 cores) processor and one graphics processing unit (NVIDIA A100 40 GB). Moreover, all steps were run on Google Colaboratory (free‐to‐use hardware).^37^ The DAE was implemented in Python with the help of the Pytorch lightning interface.^38^, ^39^ The architecture of the network and training parameters were optimized using the Bayesian Optimization HyperBand algorithm^40^ with the help of the Tune framework.^41^ The details of the optimization are given in Supporting Information Text S1. All training was performed using the mean‐squared error loss and an Adam optimizer^42^ with a batch size of 16, a learning rate of 4 × 10^−5^, and 150 epochs. The training progress for the simulated data set is provided in Supporting Information Figures S2 and S3.

An early‐stopping strategy^38^ was performed by monitoring the average error of the validation subset at the end of every epoch and stopping the training when no improvement was observed in 10 epochs. The SR and the SRF methods were tested using the FID‐A toolbox,^43^ the CrR and the CrRF methods with the Gannet toolbox,^28^ the frequency domain correlation and frequency domain correlation over a limited frequency range methods,^5^ and previous studies (Tapper et al^7^ and Ma et al^22^) with our in‐house code.

All proposed methods (dCrR, dCrRF, dSR, and dSRF) were trained, validated, and tested using the simulated and the phantom data set, and the dCrR method was trained and tested using the big GABA data sets.

### 2.6 Statistics and quality evaluation

#### 2.6.1 Performance analysis

For the simulated data set, in which the true shifts from the basis signal were known, the error was defined as the difference between the estimated and the true shifts. The accuracy and precision of an FPC method were established as the average and the SD of the error, respectively. In addition, the performance of the dCrR method trained with the simulated data set was investigated beyond the trained range of frequency and phase (−40 to 40 Hz and −180° to 180°, respectively).

For the phantom and the Big GABA data set, in which true shifts were not known, the quality of alignment was measured by comparing the similarity index (SI), which is the sum of all elements of a similarity matrix. Each element of the similarity matrix is the normalized scalar product (the equation as implemented is provided in Supporting Information Equation S1) for each pair of spectra (fast Fourier transformation of a FID) over a limited frequency range (from 2.5 to 3.5 ppm). Note that higher SI denotes superior performance.

#### 2.6.2 Performance against noise

The stability of the dCrR, dSR, and SR method against noise was evaluated. A set of transients was generated using the following procedure. First, a frequency shift of 5 Hz and a phase shift of 45° were added to $S_{\text{basis}}\left(t\right)$. Second, 20 realizations of a random complex Gaussian noise with a linearly increasing SD were introduced to the shifted signal such that SNR was in the range of approximately 8 to 110. The frequency and phase shifts of the generated set were estimated using the networks trained with the simulated data set. The FPC performance was evaluated as a function of SNR.

#### 2.6.3 Monte Carlo analysis

Monte Carlo studies were carried out to investigate the performance of the dCrR, dSR, and SR methods. A set of transients was generated using the following procedure. First, a frequency shift of 5 Hz and a phase shift of 45° were added to $S_{\text{basis}}\left(t\right)$. Second, 256 realizations of a random complex Gaussian noise with the same SD were introduced to the shifted signal such that SNR was approximately 15. The frequency and phase shifts of the generated set were estimated using the networks trained with the simulated data set. The FPC performance of dCrR, dSR, and SR methods was compared.

## 3 RESULTS

The training and processing time of each proposed network for the simulated data set is listed in Table 1. Approximately, frequency and phase estimation of one transient requires approximately 3 ms. The evaluation of the performance of different methods (precision, SI, linewidth, the processing time of FPC, and training time for DAE) for the test signals of the simulated data set and of the phantom are summarized in Tables 1 and 2, respectively.

**FIGURE 3:**
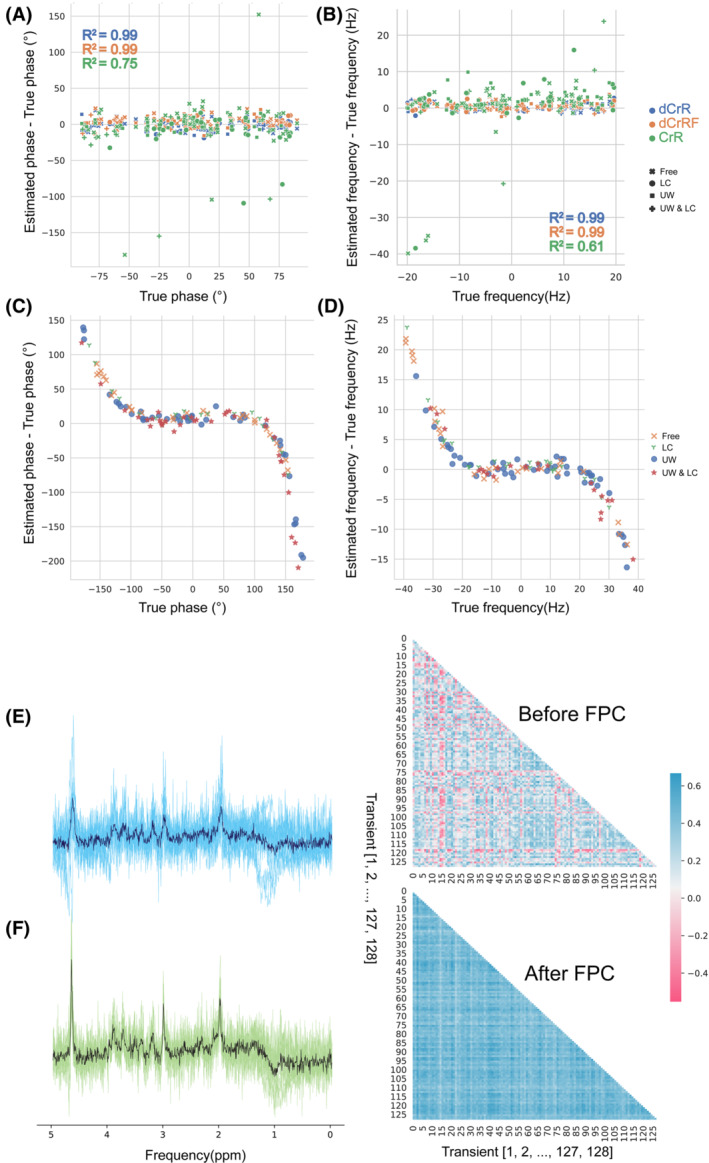
A, B, Testing results of the simulated data set. A, B, Plots of the error in deep learning (DL)–estimated phase (A) and frequency (B) shifts against the actual shifts, respectively. The R^2^ values of the method types are color‐coded. C, D, Testing the dCrR method beyond the trained range of phase and frequency (−180° to 180° and −40 to 40 Hz, respectively). Plots of the error in DL‐estimated phase (C) and frequency (D) shifts against the actual shifts, respectively, using dCrR. E, Uncorrected spectra and their similarity matrix. F, The same spectra after frequency and phase correction (FPC) using dCrR and their similarity matrix. Dark blue and green spectra show the average uncorrected and corrected spectra, respectively. Abbreviations: CrR, creatine referencing; dCrRF, dCrR over a limited frequency range; Free, without any nuisance peak; LC, with lipid peak; UW, with unstable water peak

The Bland–Altman plots in Figure 3A,B show the accuracy and biases in the estimation of frequency and phase shifts using dCrR, dCrRF, and dCr. The highest precision (0.94 Hz and 6.62°) among the absolute FPC methods was achieved with the proposed dCrR. Although dCrRF performed similarly to dCrR, CrR showed the lowest performance (7.92 Hz and 32.34°). All of the proposed methods performed well in spectra with and without the simulated nuisance peaks. The agreement, estimated by R^2^ value, was high for dCrR (*R* ^2^_frequency_ = 0.99 and *R* ^2^_phase_ = 0.99) and dCrRF (*R* ^2^_frequency_ = 0.99 and *R* ^2^_phase_ = 0.99) and moderate for CrR (*R* ^2^_frequency_ = 0.61 and *R* ^2^_phase_ = 0.75). Figure 3C,D illustrates the results of dCrR on unseen test signals in which offsets are beyond trained bound (−40 to 40 Hz and –180° to 180°). The network showed poor performance beyond its trained bound (precisions for frequency and phase estimation were 8.69 Hz and 60.04°, respectively). Figure 3E,F shows the spectra and the similarity matrix heatmaps obtained before and after the FPC tested, respectively. The dCrR method increased the SI in the visualized test signals from 0.14 to 0.42.

**TABLE 2:** Comparison of our proposed method with existing commonly used FPC methods for the phantom data set

	SI	Cr_linewidth_ (Hz)
dCrR	0.66	6.02
dCrRF	0.66	**6.00**
dSR	0.60	6.80
dSRF	**0.67**	6.03
SR	0.58	6.75
SRF	0.60	6.46
CrR	0.58	7.03

*Note*: Bold text indicates the best performance in each metric. The SI of the test subset without FPC was 0.50.

**FIGURE 4:**
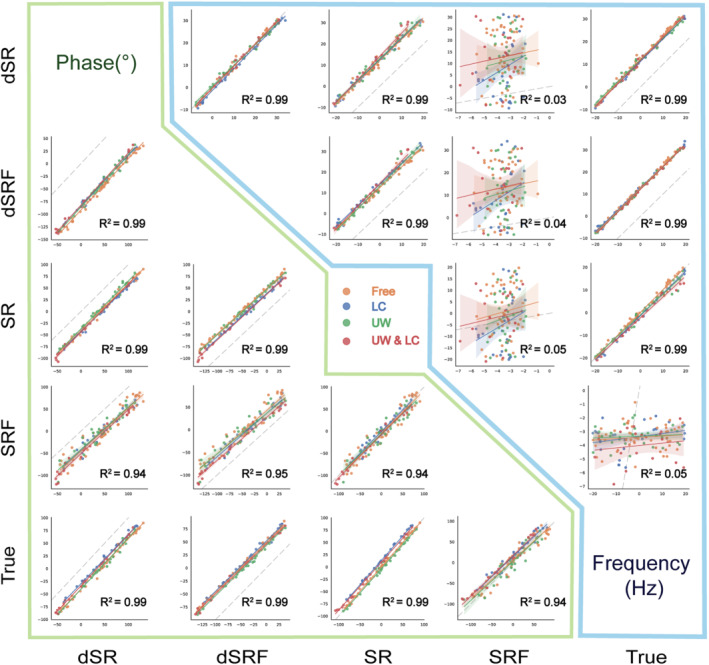
Pairwise comparison of the results of the spectral registration (SR)–based FPC methods for the test subset of the simulated data set. The upper triangular shows the correlations of frequency estimations, and the lower triangular shows the correlations of phase estimations. The fitted lines represent the linear regression model and the 95% confidence interval. The type of simulated nuisance peak is color‐coded. The total *R* ^2^ values are calculated along the corresponding axes. The dashed gray lines are identity lines. Note that the reference signals of relative FPC methods were different, which resulted in shifting their scatter plot from the identity line. Abbreviations: dSR, deep learning–based SR; dSRF, dSR over a limited frequency range; SRF, SR over a limited frequency range

Figure 4 shows a pairwise correlational comparison of the relative FPC methods (our proposed dSR and dSRF with SR and SRF). The *R* ^2^ value of each method pair and the true value of shifts are reported in the corresponding axes. The *R* ^2^ indicates that there is a high degree of agreement between the frequency and phase estimations between methods except for SRF. The agreement between the estimations of methods and true values (*R* ^2^) was high for dSR, dSRF, and SR and was low for SRF (*R* ^2^_frequency_ = 0.03 and *R* ^2^_phase_ = 0.94).

**FIGURE 5:**
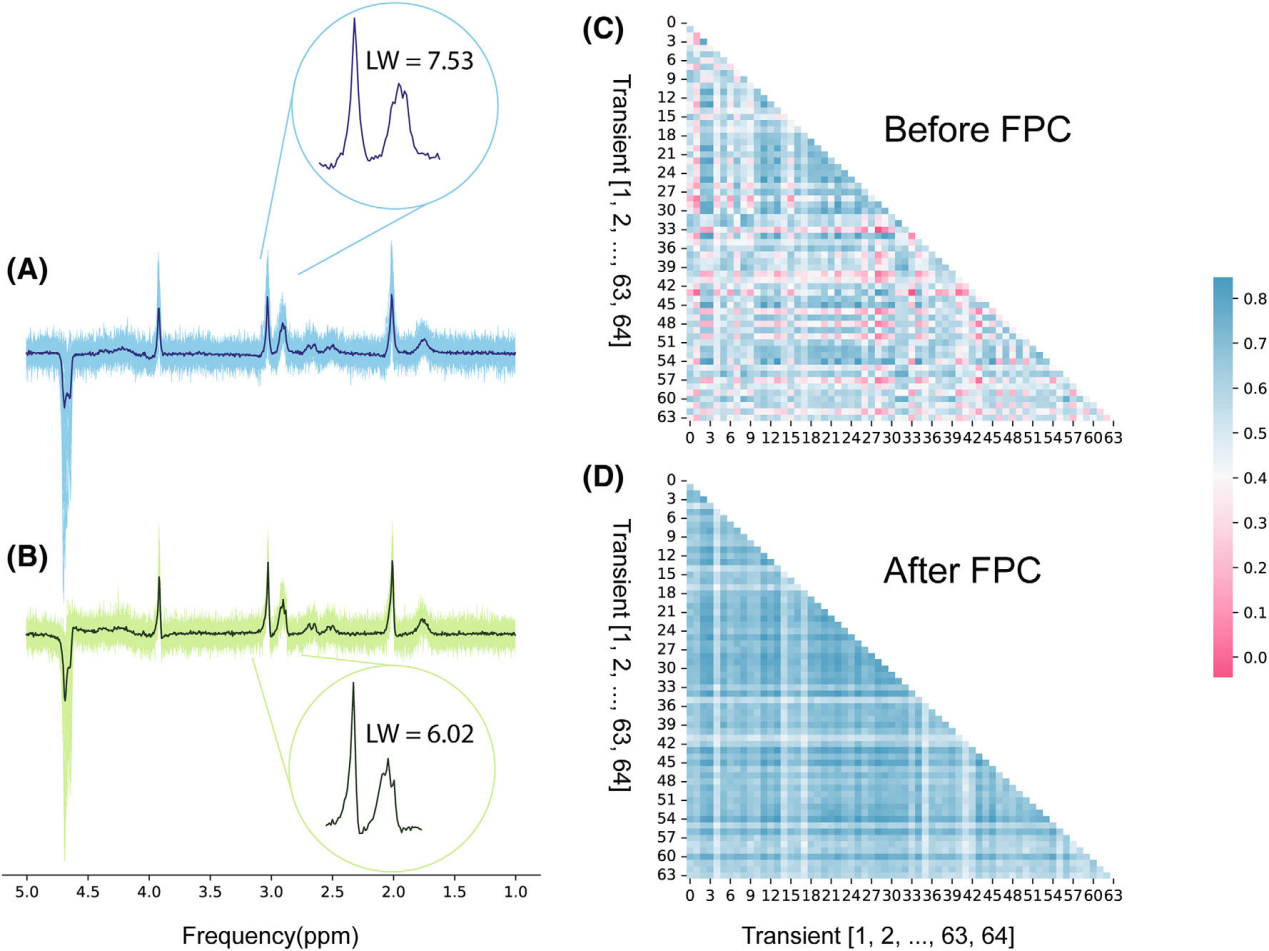
Frequency and phase correction of the phantom test subset using the dCrR method. Uncorrected (A) and corrected (B) spectra from the test subset. Dark blue and green spectra show the average uncorrected and corrected spectra, respectively. The circled inset shows the zoomed creatine (Cr) peak at 3 ppm. The similarity matrix of 64 samples of the test subset before (C) and after (D) FPC

Figure 5 shows spectra from the test signals of the phantom data set and the corresponding heatmaps of similarity matrices before and after correction using the dCrR method. The dCrR method achieved the highest performance and increased the similarity among transients from 0.50 to 0.66, and decreased the linewidth of Cr peak at 3 ppm in the averaged spectrum by 1.5 Hz (Table 2). We observed that changing temperature during measurement altered the amplitude and linewidth of peaks, which resulted in a decreased similarity index. The dSR method failed to increase the SI and reduce the Cr linewidth because dSR tends to correct frequency and phase shifts of large frequency components, such as the residual water. However, dSRF overcame this issue and demonstrated superior performance by limiting the frequency range.

**FIGURE 6:**
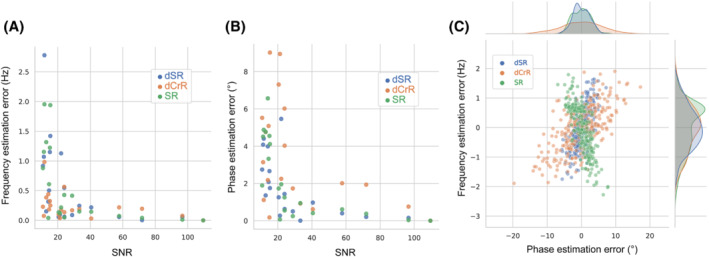
A, B, Comparison of frequency (A) and phase (B) correction precision of the dSR, dCrR, and SR methods over various SNR levels. C, The results of Monte Carlo (MC) analysis. Comparison of dSR, dCrR, and SR methods. For the sake of visualization of absolute and relative methods alike, the estimations from each method were subtracted from their average value. The results of the dCrR method without subtracting from its average value can be found in Supporting Information Figure S1

The link between the absolute error in estimates and the SNR is shown in Figure 6A,B using a scatter plot. The comparison of the proposed dSR and dCrR with SR methods is presented. In low SNR, dSR and SR methods outperformed dCrR in terms of the precision of phase shift estimation, but dCrR demonstrated more resilience in terms of the precision of frequency estimation.

Figure 6C shows a comparison of the dSR, dCrR, and SR methods in the Monte Carlo analysis using a scatter‐plot visualization of the joint distribution of frequency and phase. For the simulated data set, the mean error of dSR (1.36 ± 0.69 Hz and 0.51 ± 2.33°) and SR (−0.67 ± 0.81 and 3.15 ± 2.53°) showed similar performance, whereas dCrR performed less precise phase‐shift estimation (4.69 ± 0.77 Hz and − 41.27° ± 5.827°). The true values of the frequency and the phase shifts were 5 Hz and 45°.

**FIGURE 7:**
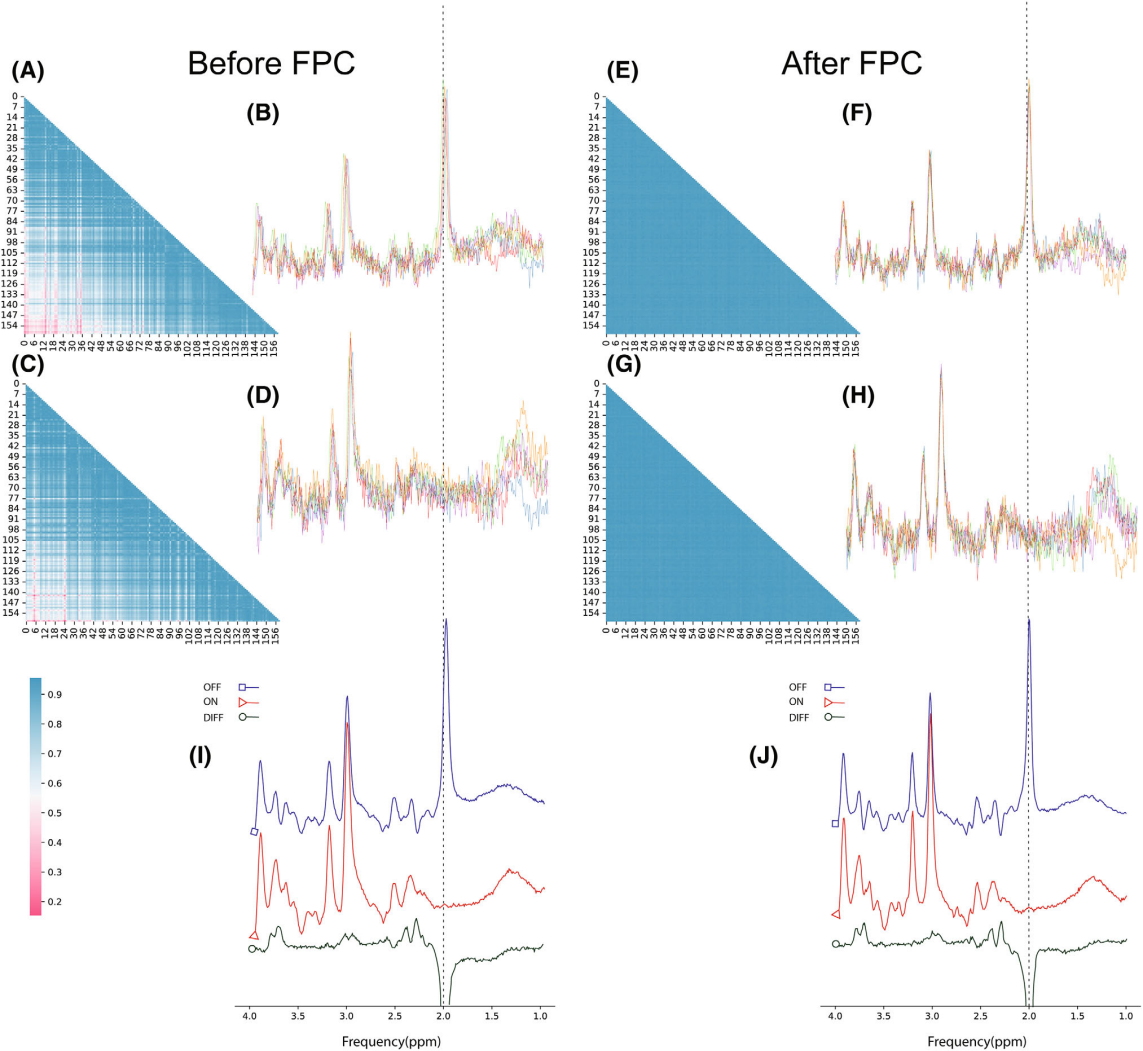
An example of FPC using dCrR for a test set in the GABA‐edited in vivo data set. Unedited spectra (A) and their similarity matrix (B) before FPC. Edited spectra (C) and their similarity matrix (D) before FPC. Unedited spectra (E) and their similarity matrix (F) after FPC. Edited spectra (G) and their similarity matrix (H) after FPC. (I) Average uncorrected spectra (blue, unedited; red, edited) and their difference (dark green). (J) Average corrected spectra using dCrR (blue, unedited; red, edited) and their difference (dark green)

Figure 7 illustrates an unseen test subset of a GABA‐edited in vivo data set (site = 1, subject = 3; 160 edited transients; 160 unedited transients) and a heatmap of their similarity matrix before and after FPC by the dCrR method. Our method increased the SI in the visualized test subset from 0.80 and 0.82 to 0.92 for edited (ON) and unedited (OFF) spectra, respectively. In all test subsets, the SI was increased from 0.88 ± 0.05 to 0.93 ± 0.02.

**FIGURE 8:**
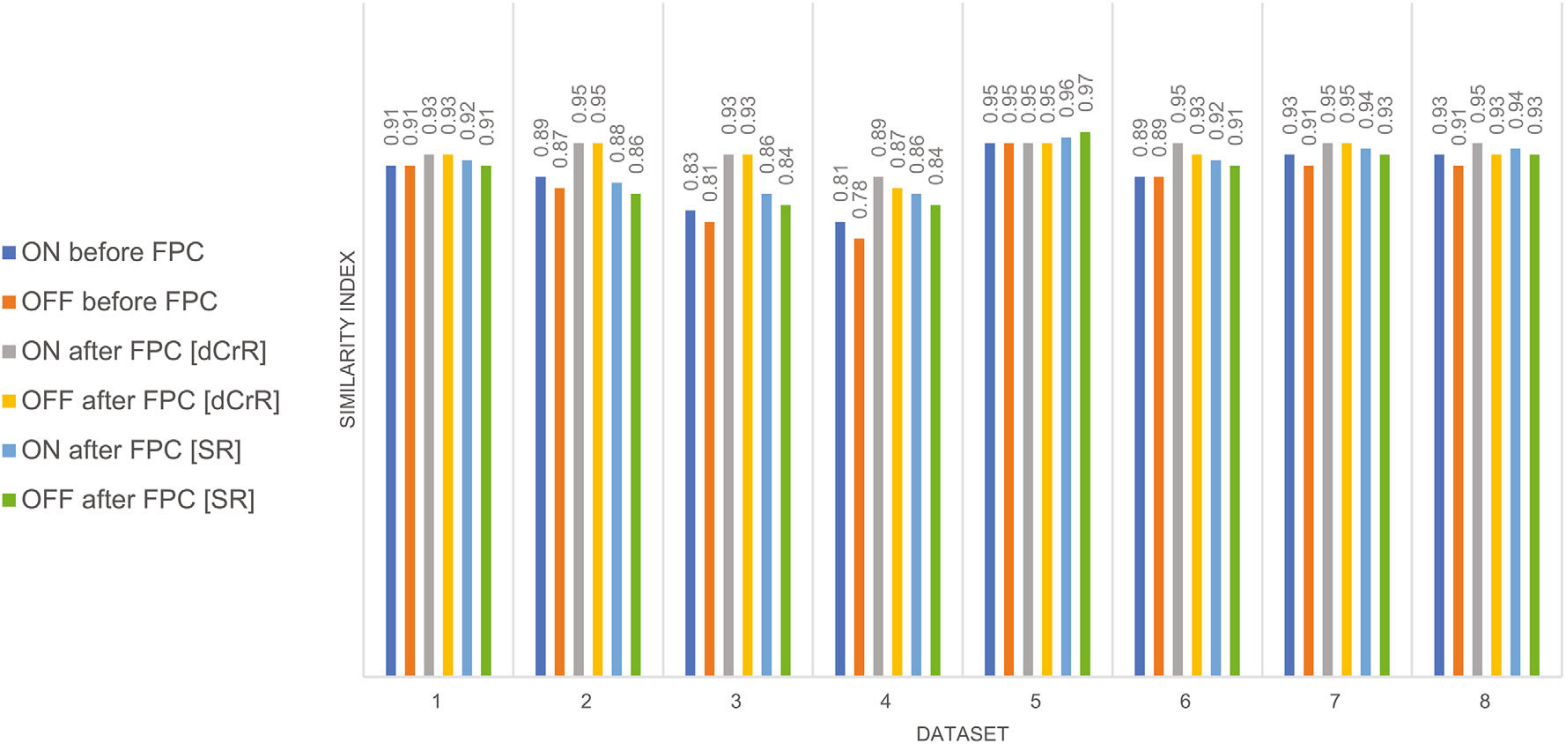
Similarity index comparing dCrR to SR for test signals of the Big GABA data set

Figure 8 shows the comparison of the results of dCrR‐based and SR‐based correction of ON and OFF transients of the test subsets. On average, dCrR performed better than SR, as indicated by the mean SI (the mean SI was increased from 0.88 ± 0.05 [ON: 0.89 ± 0.04, OFF: 0.87 ± 0.05] to 0.93 ± 0.02 [ON: 0.93 ± 0.02, OFF: 0.93 ± 0.02] and 0.90 ± 0.04 [ON: 0.91 ± 0.03 OFF: 0.89 ± 0.04] by dCrR‐based correction and SR‐based correction, respectively).

## 4 DISCUSSION

In this study, the combination of DL and mathematical modeling was demonstrated to be able to provide FPC in simulated, phantom, and in vivo MRS data. We compared our method with five previously published methods: SR, SRF, frequency domain correlation, frequency domain correlation over a limited frequency range, and CrR, as well as supervised DL‐based methods. We evaluated the ability of each of these methods to estimate the frequency and phase shifts in the simulated MRS data set with known shifts and at varying SNR levels. Our results (Table 1) indicated an improvement in performance in terms of precision and the SI, the latter of which is a new measure proposed in this study for evaluating the FPC performance. Additionally, we compared our results with those obtained using other methodologies in terms of the linewidth of the Cr peak of the averaged signal obtained by summing corrected transients, and we discovered that the unsupervised DL‐based methodology performed comparably to others.

We found that the traditional FPC over a limited frequency range performed poorly in spectra with low SNR (Table 1), whereas our proposed FPC methods over a limited frequency range (dSRF and dCrRF) can perform equally (Table 1) to our proposed methods operating in the time domain (dCrR and dSR). Our methods also performed well in a Monte Carlo study, in which the phase‐shift and frequency‐shift estimate precisions were found to be very good and in general accordance with the SR method (Figure 6C).

While the performance of conventional SR‐based FPC methods can depend on the presence of nuisance peaks (Table 1 and Refs 3, 7), our result demonstrated that our methods functioned effectively in the simulated data set regardless of the presence of nuisance peaks (Table 1).

Our proposed method can be trained in a few minutes (Table 1) because of using a one‐dimensional signal as the input and a relatively tiny network. In the case of large MRS data sets or repeated measurements with the same conditions, the training time will be compensated by the FPC processing time, which is significantly shorter than in methods based on nonlinear least squares (Table 1). Moreover, the training time can be reduced by using well‐established methods such as few‐shot transfer learning,^13^, ^44^ in which a network pretrained on large simulated or in vivo data sets can be trained in a few iterations using a few samples.

When the SNR of the test signals was lowered, the performance of our proposed methods (dCrR and dSR) and SR was reduced. When the SNR was decreased below the SNR of the training set, the performance deteriorated even further, as the signal is dominated by noise. We observed that in low‐SNR signals, the dSR and SR methods performed better in phase‐shift estimation, whereas dCrR worked much better in frequency estimation (Figure 6).

Regardless of performance, the encoder/model‐decoder provides a unique flexibility advantage. It leverages the underlying prior knowledge, which can be beneficial for estimating frequency and phase, independent of the kind of MRS data. Therefore, this approach may be used immediately to almost any sort of MRS data with little or no modification.

Contrary to the previous applications^7^, ^22^ of DL in FPC that used a supervised way utilizing simulated data, our proposed network was trained in an unsupervised way. This is advantageous, as most of the MRS data are unlabeled, and simulated data sets may not accurately reflect all in vivo circumstances, such as macromolecules and artifacts. Additionally, a single network was trained in this work to deliver both frequency and phase estimations by including prior knowledge into the decoder, whereas earlier work failed to train a single network.

It has been demonstrated that traditional FPC methods can benefit from additional information in a data set,^10^ addressing the problem of selecting a reference signal in SR by using a weighted average reference determined by mutual information in data. The encoder/model‐decoder may assist in extracting patterns and information from data by introducing more complex models.

Overfitting is a common pitfall in DL.^13^ We implemented a dropout layer in the input to remove a part of the input randomly in every training step, which is a computationally inexpensive and very effective regularization strategy for decreasing overfitting and increasing the generalization of the network.^32^

Training neural networks for regression problems necessitates a well‐calibrated estimation. The results revealed a significant linear link between the true and estimated values (Figures 3 and 4), indicating a well‐calibrated estimation, although additional examination of the results is necessary.

Along with demonstrating the performance of the proposed approach on simulated and phantom data, the method was used to carry out FPC and enhance the similarity of signals in publicly accessible GABA‐edited in vivo MRS data. It should be emphasized that the proposed network was fed with both edited and unedited signals and trained simultaneously. The result shows the same performance for the edited and the unedited input.

In general, DL‐based techniques are restricted in terms of generalizability,^13^ especially DL algorithms in the MR domain due to the fact that their training data might be confined to a single scanner, a single sequence, and/or a single vendor.^45^

In this study, only phantom data and in vivo data sets gathered from four distinct locations utilizing a single vendor and a single sequence were used to demonstrate the applicability of our method. A crucial step toward the generalizability and clinical use of our method is training and testing using multicenter and multivendor data.

One caveat in this study is that comparing processing time per transient between algorithms might not be widely valid, as it might be affected by the parameters and conditions of algorithms. Using FLOPS (floating‐point operations per second) to assess the computing cost^46^ can help for a better comparison.

In the present study, we focused primarily on the validation of our proposed methods using in silico ground‐truth knowledge and showed its application in GABA‐edited in vivo MRS data. We are aware that further evaluation and comparison with a more robust method^10^ are needed. In addition, Cr referencing‐based approaches, such as dCrR, are not the optimal way for GABA‐edited in vivo MRS data alignment,^47^ and dSR or a more sophisticated DL‐based method should be investigated in future researches.

## 5 CONCLUSIONS

In general, our proposed time‐domain FPC method, which is based on DL networks trained in an unsupervised way with complex data, may yield results comparable to previous FPC methods. The proposed approaches can perform absolute and relative FPC on extensively manipulated data in a shorter amount of time once the network is trained. Thus, our proposed approach could aid in the acceleration of analyzing large MRS data sets. Further study is needed to evaluate the generalizability of the proposed methods for multivendor data.

## FUNDING INFORMATION

This work is part of the project that has received funding from the European Union's Horizon 2020 research and innovation program under the Marie Sklodowska‐Curie grant agreement No 813120 (INSPiRE‐MED) and has been also supported with the institutional support RVO:68081731 ‐ Czech Academy of Sciences, Institute of Scientific Instruments.

## Supporting information


**Figure S1** Bin‐based visualization of the result of the Monte Carlo analysis of the simulated data set
**Figure S2** Online monitoring of precision of a set of transients in the validation subset during training
**Figure S3** Validation loss (mean square error [MSE]) versus training steps
**Figure S4** Process flow of one‐shot frequency and phase correction of J‐difference‐edited MR spectra using a single deep neural network
**Figure S5** Illustration of the proposed convolutional encoder–model decoder for deep learning–based spectral registration (dSR) method
**Figure S6** Reference scan selected by our proposed machine learning–based algorithm for the deep spectral registration method
**Table S1** Parameters for generating the simulated nuisance peak
**Table S2** Summary of the encoder's network
**Text S1** Bayesian hyper‐parameterization
**Text S2** A novel machine learning–based algorithm for finding the reference scan for the dSR method
**Equation S1** Equation for deriving each element of the similarity matrix

## Data Availability

The source code is freely available at https://github.com/isi‐nmr/DeepFPC. For questions, please contact the authors. For testing the frequency domain correlation and frequency domain correlation over a limited frequency range, we developed a *MATLAB* script, which is publicly accessible at https://github.com/isi‐nmr/Frequency‐and‐Phase‐Correction‐of‐MRS‐signals‐Using‐Cross‐Correlation.
